# Is protease inhibitors based antiretroviral therapy during pregnancy associated with an increased risk of preterm birth? Systematic review and a meta-analysis

**DOI:** 10.1186/s12978-016-0149-5

**Published:** 2016-04-05

**Authors:** Yonatan Moges Mesfin, Kelemu Tilahun Kibret, Amsalu Taye

**Affiliations:** Department of Public Health, College of Medical and Health Sciences, Wollega University, Nekemte, Ethiopia; Department of Public Health, College of Medical and Health Science, Haramaya University, Harar, Ethiopia

**Keywords:** Risk factors, Antiretroviral therapy, HIV, Pregnancy, Prematurity, Protease inhibitors, Preterm birth

## Abstract

**Background:**

Antiretroviral therapy is recommended during pregnancy to decrease the risk of perinatal transmission of HIV-1 infection and to improve maternal health. However, some studies have reported that antiretroviral treatment (ART) containing protease inhibitors (PI) is associated with an increased risk of preterm birth. In contrast, other studies have reported no increased risk. This meta-analysis was conducted to derive a more reliable estimate of the association between the prenatal use of PI based ART regimen and preterm birth.

**Methods:**

A systemic review and meta-analysis was conducted using published studies which were identified through a computerized search using the Medline/PubMed database, Google Scholar and Health Inter Network Access to Research Initiative (HINARI). The analysis was undertaken using STATA version 11.0 software and studies were described by forest plot. Heterogeneity across studies was checked using Cochran Q test and I^2^ test. An adjusted odd ratio with 95 % confidence intervals [95 % CI] was pooled using a random effects model.

**Results:**

The Cochrane Q test (Q test *p* = 0.051) showed a good homogeneity among studies. However, medium heterogeneity was observed in up to 46 % of the sample using the I^2^ test (I^2^ = 46.5 %). The Egger weighted regression method (*p* = 0.04) showed evidence of publication bias, but Begg rank correlation statistics (*p* = 0.47) did not show evidence of publication bias. The pooled analysis of 10 studies showed that protease based ART exposure during pregnancy was associated with an increased risk of preterm birth (pooled odds ratio 1.32 (95 % CI, 1.04 to 1.59).

**Conclusions:**

This meta-analysis revealed that the PI based ART exposure during pregnancy is significantly associated with an increased risk of preterm birth. There should be strong cautions against initiating ART during pregnancy and PI based ARV should be replaced by others drug regime. Protease inhibitor ART drugs should not be included as part of therapy during pregnancy.

## Background

The introduction of antiretroviral agents has been successful in reducing of human immune virus (HIV) transmission from mother to baby [1–5]. Antiretroviral therapy (ART) is recommended during pregnancy to decrease the risk of perinatal transmission of HIV-1 infection and to improve maternal health [6]. Pregnant women with symptomatic HIV [7], high viral loads, and low CD4+ cell counts are more likely to be treated with highly active antiretroviral therapy (HAART), including ART with protease inhibitors (PIs) [8]. The World Health Organization (WHO) now recommends HAART as an option of mother to child transmission (PMTCT) during pregnancy for all HIV-infected women with CD4 cell counts less than 350 cells/mm3 [1].

Safety concerns have been raised about the use of PI antiretroviral agents [9]. A systemic review revealed that low birth weight (LBW) and preterm birth (PTB) were observed in women receiving PI based ART. Treatment regimens including protease inhibitor and timing of ART initiation (at early pregnancy) are factors associated with these adverse pregnancy outcomes [10].

These uncertainties, especially the association between PI-based ART regimens and preterm births, remain unresolved. Other studies exploring the association between ART including PIs and preterm birth [7–9, 11–19] had shown conflicting results. Some of these observational studies found an increased risk of preterm birth [7, 8, 11, 12, 15–20] while other studies have reported no increased risk of preterm birth [9, 13, 14].

It is crucial to evaluate the effect of PI-based ART exposure during pregnancy and the risk of prematurity/preterm births to solve these controversies. Therefore, we conducted a meta-analysis of all the relevant studies published to date in order to provide a more reliable overall estimate of the association between prenatal uses of PI-based ART regimens and prematurity. This paper summarizes the evidence of the association between PI-based ART regimens and preterm births through a systemic review and meta-analysis of studies.

## Methods

### Study design and data source

We assessed and analyzed available evidence on the following question: does the use of any PI based antiretroviral drugs by HIV infected women that was started at any appoint during pregnancy or preconception lead to an increased risk of prematurity? We defined prematurity as a newborn of less than 37 completed weeks of gestation, in HIV-infected women. A systematic review and meta-analysis of studies that reported the association between PI based ART and PTB was conducted. We identified English language publications in the Medline data base, Google Scholar and HINARI (Health Inter Network Access to Research Initiative). We also cross-checked the reference lists with combinations of the key wards “risk factors”, “antiretroviral therapy”, “HIV”, “pregnancy”, “prematurity”, “protease inhibitors”, “preterm delivery” and “low birth weight”. These mesh terms were used as a combination of free text and thesaurus terms in different variations. The review and data abstraction was performed from November 1, 2014 to February, 2015.

### Study selection

A systemic review and meta-analysis was conducted on studies that reported the association between PI based ART and preterm birth. Studies were selected for the meta-analysis if they included an appropriate control group for comparison (defined as a group of HIV-infected women receiving antiretroviral agents without PI during pregnancy), so that the effect of antiretroviral agents on preterm delivery could be compared with a group of HIV-infected women receiving PI based ART therapy. We included studies where the women initiated ART before and during pregnancy (in the first, second or third trimester). The studies also presented adjusted effect estimates in odds ratios, rate ratios, risk ratios and hazard risk ratios.

Reports of original studies, unpublished master’s thesis and PhD dissertations, which were written in English also considered.

Commentary, editorials and reviews were excluded. In addition, studies were excluded from the analysis for any of the following reasons: articles that did not focus on PI based ART, those that did not consider PI based ART as risk factors (independent variable), and studies that did not give effect estimates as odds ratios, rate ratios, or risk ratios, or did not allow the computation of such measures. Duplicate publication of the same study, articles available only in abstract form, articles with greater than 20 % lost during follow up or non-response were also excluded. The selection of articles for review was done in three stages: titles alone, abstracts, and then full text articles.

### Methodological quality assessment

The following points were noted as the study quality indicators: the use of right statistical measurement to assess the association between preterm birth and PI based ART exposure during pregnancy; assessment and adjustment of potential confounders (demographic, socio-economic and baseline characteristics of the study population). Reporting of response rates, lost to follow up and appraisal of internal validity of the study were also considered as quality indicators.

All assessments were entered into pre-tested and standardized data extraction form. Studies were assessed for quality in which studies with medium (fulfilling 50 % of the quality assessment parameter) and high quality were included for analysis. The studies that reported outcomes on at least 50 patients, the studies whose response rate was greater than 80 %; those that reported basic demographic data, and adjustment for covariates like demographic, socio-economic and baseline characteristics of the study population were considered as high quality studies.

#### Data abstraction

The data abstraction was conducted independently by two investigators (KT, AT) and cross checked by the third researcher (YM). The selected studies were reviewed using a pretested and standardized abstraction format and the following data were extracted, title, authors name, year published, country, study design, study site/country of origin, sample size, response rate, other factors adjusted for in the analysis, intervention (type of antiretroviral therapy used, time of initiation during pregnancy or before pregnancy), premature delivery definition and rate, and measure of rate with its confidence interval (CI). In order to standardize the results, all measures of effect were expressed as OR, and a 95 % CI was calculated if this had not calculated. When there was a discrepancy in data abstraction among the investigators, it was resolved through discussion and consensus.

### Statistical analysis

Epi data version 3.1 and STATA version 11 software were used for data entry and analysis respectively. The descriptions of original studies were made by tables and forest plots. The pooled effect size of PI based ART exposure on preterm birth was carried out using the Der Simonian-Laird random-effects meta-analysis (random effects model) and measured by odds ratio with a 95 % confidence intervals [95 % CI].

### Statistical heterogeneity and exploration of publication bias

Statistical heterogeneity was evaluated by Cochran’s Q test. This shows the amount of between study heterogeneity and I^2^ statistic. The I^2^ statistic is a measure of the percentage of variability (inconsistency) between studies that is due to chance. The presence of statistical heterogeneity was tested using Cochran’s Q test (*p* < 0.10 is considered statistically significant) and I^2^ test (values of 25 %, 50 % and 75 % are considered to represent low, medium and high heterogeneity respectively) [21].

The individual study odds ratio (OR) with 95 % confidence intervals (CIs) were displayed using Funnel plots to assess publication bias. The Egger weighted regression and Begg rank correlation tests were used to assess publication bias (*p* < 0.05 is considered statistically significant). Cumulative meta-analysis was also used to explore the effect of each study and less precise studies on the pooled estimates.

#### Ethical issues

Since this research is a systemic review and meta-analysis ethical approval is not necessary, thus it was not sought.

## Results

### Identified studies

**Fig. 1:**
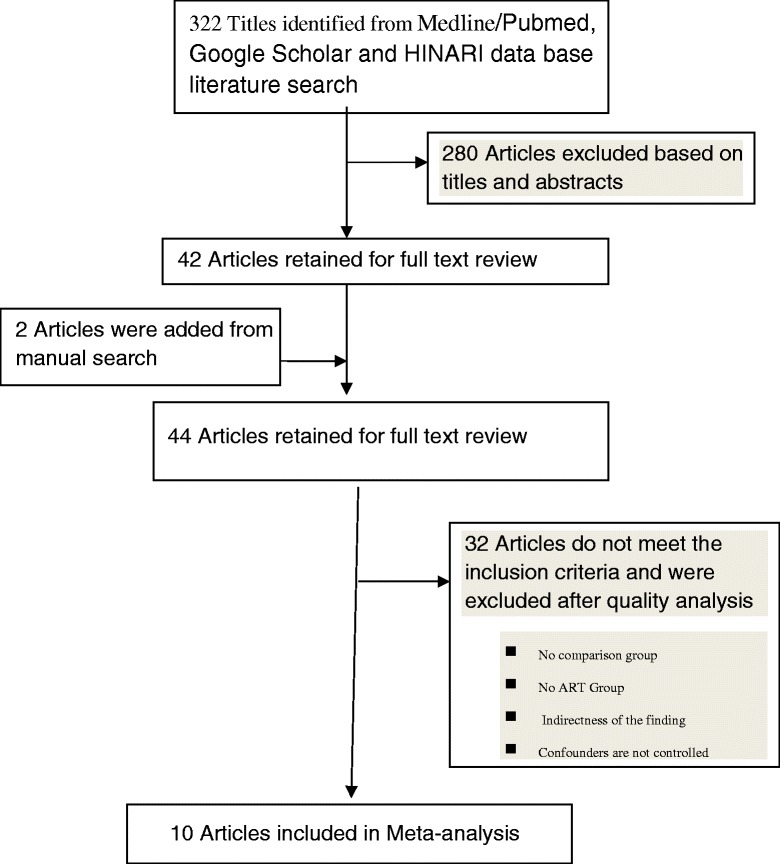
Flow chart diagram describing selection of studies for a systematic review (identification, screening, eligible and included studies). Articles may have been excluded for more than one reason

A total of 322 articles were identified through the literature search. Two additional articles were also obtained manually. Of these, 290 were excluded after screening by titles and abstracts. These were duplicated studies, case reports, systematic reviews and meta-analyses. From the remaining 44 articles, 32 were excluded because of one or more of the following reasons, they were studies of PMTCT, they did not consider PI based ART as an independent/exposure variable, the full article or data could not be accessed, they were on children, they were dealt only with monotherapy, or they did not give a quantitative effect estimate. Further studies were excluded that did not adjust for covariates. Finally 10 articles were used for the meta-analysis. See Fig. 1 for the flow diagram for study selection.

### Study characteristics

Eight of the 10 studies selected for the meta-analysis were prospective cohort studies [8, 11–15, 17, 22], one study was a randomized trial study [23] and there was one surveillance study [16]. These 10 selected studies addressed the association between PI based ART and preterm birth, and had sample size ranging from *n* = 183 in Germany and Austria [15] to *n* = 5009 in UK [16]. The studies were carried out between 2002 [22] and 2013 [13]. All studies were reported in English. These studies represented 19 countries across 5 continents (Africa, Europe, Latin America, and North America). General characteristics and a description of the studies selected are available in Table 1.

**Table 1:** Summary of the 10 observational studies assessing the association between Protease Inhibitors based ART and preterm birth included in the meta-analysis

First author, ref year	Design	Sample size	Setting/country	Comparison regime	Time of initiation during pre/before pre	PTD type	AOR (95 % CI)
Cotter AM et al., 2006 [8]	Prospective	999	Miami, Florida, USA	Combination without PI	<12wk,12–23wk, 3rd trimester	<37wk	1.8 (1.1,3.0)
Powis KM, et al., 2011 [23]	Randomized	730	Botswana	Triple NRTI based ARTwout PI	Between 26 and 34 weeks	<37wk	2.02 (1.25–3.27)
Grosch-Woerner I et al., 2007 [11]	Prospective	183	Germany & Austria	Mono therapy	>2wks before conception) or during pregnancy	<36wk	3.40 (1.13–10.2)
Schulte J, et al., 2012 [12]	Prospe	8793	Atalanta, Georgia	2drugs	26–42wk	37	1.21 (1.04–1.40)
Watts DH et al., 2013 [24]	Cohort	1869	USA	Mono or dual therapy	1st -3rd trimester	37	1.49 (0.83,2.66)
Tuomala RE et al., 2002 [13]	Trial & cohort	2123	Boston USA	Combination without PI	1st -3rd trimeste	37	1.80 (0.94–3.43)
Patel K et al.,2011 [22]	Prospe cohort	777	USA	Mono/combination without PI	1st -3rd trimeste	37	1.29 (0.77, 2.15)
Szylda EG, et al., 2006 [14]	Prospective cohort	681	Argentina, Bahamas,Brazi, Mexico	1–2 NRTI	Before/during pregnancy	37	1.1 (0.5–2.8)
Townsend CL et al., 2007 [15]	Surveillance	5009	UK	Combination	Before/during pregnancy	37	0.96 (0.78,1.19)
Hankin C et al., 2003 [16]	Prospecti	2326	9 EU country	No ART	>>	37	4.14 (2.36,7.23)

### Assessing heterogeneity and publication bias among the studies

**Fig. 2:**
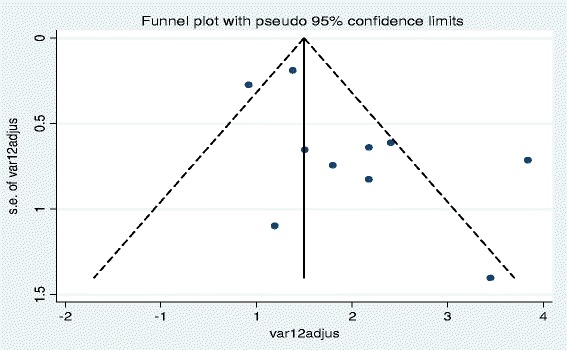
Funnel plot of with 95 % confidence limit; the horizontal line in the funnel plot indicates the effect estimate, while the sloping lines indicate the expected 95 % confidence intervals

**Fig. 3:**
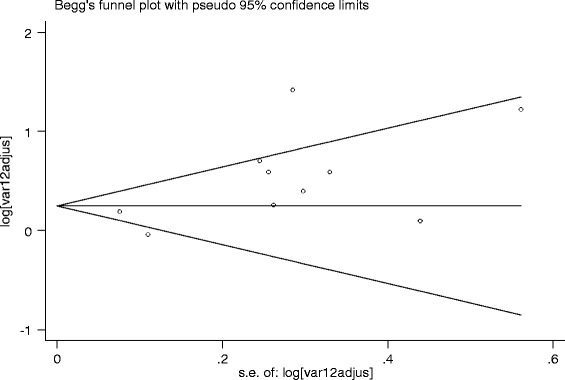
Begg’s funnel plot with 95 % confidence limit; the horizontal line in the funnel plot indicates the natural logarithms of the effect estimate, while the sloping lines indicate the expected 95 % confidence intervals

The studies showed heterogeneity using Cochrane Q test statistic (Q test *p* = 0.051). Medium heterogeneity was observed up to 46 % using the I^2^ test (I^2^ = 46.5 %) which was indicative of using random-effects model. The asymmetrical distribution of effects estimate using traditional funnel plot (Fig. 2), Begg’s funnel plots (Fig. 3) and the Egger weighted regression method (*p* = 0.04) showed evidence of publication bias. However, Begg rank correlation statistics (*p* = 0.47) showed no evidence of publication bias.

### The Association of PI based ART during pregnancy and preterm birth

**Fig. 4:**
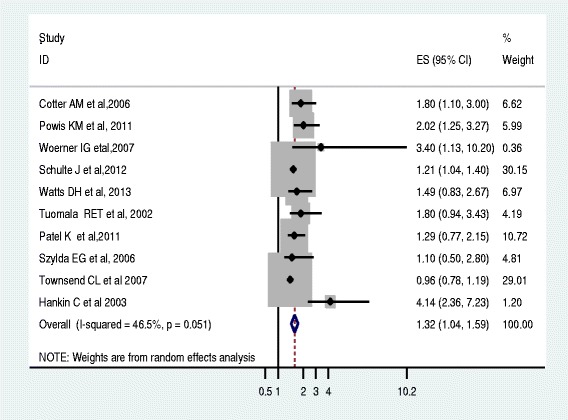
Forest plot of the 10 observational studies that quantitatively assessed the association between PI based ART and preterm birth. Size of the square is proportional to the precision of the study-specific effect estimates, and the bars indicate the corresponding 95 % CIs. The diamond is centered on the summary OR of the studies, and the width indicates the corresponding 95 % CI

Based on the ten studies that reported premature delivery in association to the use of PI based ART regimens during pregnancy, the pooled odd ratio according to random effect model was 1.32 (95 % CI 1.04 to 1.59). This meta-analysis revealed that the PI based ART is associated with an increased risk of premature births (Fig. 4).

**Fig. 5:**
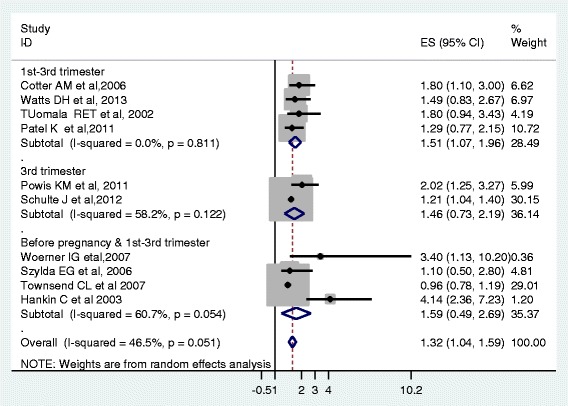
Forest plot of the 10 observational studies that quantitatively assessed the association between PI based ART and preterm birth by time of ART initiation during pregnancy

Based on the sub group analysis, we found moderate heterogeneity of effects estimate from studies that initiated ART during 3rd trimesters between study variance accounted for 58 % and 60 % before pregnancy & between the 1st and 3rd trimesters. The low heterogeneity of effect estimate (I^2^ = 0 %) of the total variance among studies which initiated ART between 1st and 3rd trimesters was noted. The pooled effects estimate for studies that initiated ART between the 1st and 3rd trimesters (4 studies) was 1.51 (95 % CI 1.07, 1.9). The pooled effects estimate for studies that initiated ART during 3rd trimester (2 studies) was 1.46 (95 % CI 0.73, 2.1). Likewise, the pooled effects estimate for studies that initiated ART before pregnancy & between the 1st and 3rd trimesters (4 studies) was 1.59 (95 % CI 0.49, 2.6) (Fig. 5).

## Discussion

The advent of ART has been successful in saving many lives [1, 5]. In the era of zidovudine and combination therapy, in utero transmission of HIV has been drastically reduced. It is evident that many children are born HIV free due to ART [2, 3]. Despite the fact that ART prevents HIV transmission from mother to fetus, several studies have reported that ART was associated with a risk of premature birth. Some studies linked the PI based ART with PTB [4, 8, 11, 12, 15, 16, 23], whereas other studies have reported PI-based ART presents no risk for PTB [13, 14, 22, 24].

This meta-analysis used 10 studies that reported the association between PI based ART and PTB in order to explore this disparity. Six of the identified studies reported that PI based ART was significantly associated with a higher risk of preterm birth [8, 11, 12, 15, 16, 23]. However, four studies revealed no significant increased risk of preterm birth linked with PI based ART [13, 14, 22, 24]. This meta-analysis combined these studies found that the PI based ART is significantly associated with an increased risk of preterm birth. According to the results of this meta-analysis, the risk of experiencing PTB by those who received PI based ART regimens was higher by 32 %. This result is consistent with a meta-analysis conducted in 2007 [9] even though its result was pooled by using unadjusted OR. Likewise, a European collaborative study across 9 European countries showed a higher risk of preterm birth among women who had received PI based ART compared with no ART [16]. This might be linked to the fact that a combination therapy with PI had been prescribed for the advanced and complex conditions, thus mothers might have been in complicated status and then their fetus would be affected.

This increased risk could be confounded by different factors other than the treatment under consideration. However, the included articles tried to adjust for several variables. The European Collaborative Study adjusted for maternal CD4 T-cell count, race, age and illicit drug use; the US studies by Tuomala et al. [17] and Cotter et al [21] also adjusted for previous premature delivery and the use of alcohol and tobacco. The study by Cotter et al. [21] also adjusted for the year of delivery and the duration of antiretroviral therapy during pregnancy. However, other unconsidered variables might have an effect on PTB. Therefore, this result should interpreted cautiously. However, this result has shown the implication of PI based ART on the normal growth of fetus. On the other hand, these PI drugs are taken as a second options for prescription and precaution have to be taken to be used by the pregnant mothers. Physicians in charge of the care of HIV positive pregnant women should substitute PI regimens by using alternative drugs.

This meta-analysis, unlike previous reviews [9], includes 10 recent studies which had adjusted OR that examined the association of PI based ART and preterm birth as well as generating the overall pooled summary estimates regarding the overall association between PI-based ART and PTB. Additionally this meta-analysis documented no significant heterogeneity among the studies, but this was not stated in the previous meta-analysis.

### Limitations

In general, the result of systematic reviews and meta-analysis needs to be interpreted in the context of the study methodologies. It is possible that confounders other than ART exposure may be responsible for the observed differences in the reported outcomes. However, we mostly selected studies that stated and accounted for possible confounders. Another likely source of bias in this review may be publication bias. Studies which find no, or negative associations, may be less likely to be published either because they are not submitted for publication, or because journals are less likely to publish them.

There are several potential limitations to this study. The analysis was based on estimates derived from observational studies that are vulnerable to confounding variables. To address the issue of potential confounders, a sensitivity analysis was performed; separate summary estimates were reported for the studies that adjusted for important potential confounders and those that did not.

## Conclusion

This meta-analysis revealed that PI based ART exposure during pregnancy is significantly associated with an increased risk of preterm birth. There should be strong cautions in initiating PI based ART during pregnancy and should be replaced by others. We conclude that the proteinase inhibitor ART drugs should not be included as part of first-line therapy during pregnancy.

## Abbreviations

AIDS: acquired immune deficiency syndrome
ART: antiretroviral therapy
HAART: highly active antiretroviral therapy
HINARI: Health Inter Network Access to Research Initiative
HIV: human immune deficiency virus
LBW: low birth weight
OR: odd ratio
PIs: proteinase inhibitors
PMTCT: prevention of mother to child transmission
PTB: preterm birth
WHO: World Health Organization
